# Progress in TLE treatment from 2003 to 2023: scientific measurement and visual analysis based on CiteSpace

**DOI:** 10.3389/fneur.2023.1223457

**Published:** 2023-10-03

**Authors:** Zhan Cao, Mingjie Guo, Xun Cao, Tiantian Liu, Shaowen Hu, Yafei Xiao, Min Zhang, Hengfang Liu

**Affiliations:** ^1^Department of Neurology, The Fifth Affiliated Hospital of Zhengzhou University, Zhengzhou, China; ^2^Department of Thoracic and Cardiovascular Surgery, The First Affiliated Hospital of Henan University, Kaifeng, China; ^3^Medical College of Zhengzhou University, Zhengzhou, China; ^4^Department of Neurology, The First Affiliated Hospital of Zhengzhou University, Zhengzhou, China; ^5^Department of Urinary Surgery, Huaihe Hospital of Henan University, Kaifeng, China; ^6^Department of Gastrointestinal Surgery, Huaihe Hospital of Henan University, Kaifeng, China

**Keywords:** temporal lobe epilepsy, treatment, Citespace, surgery, hot spot, trend

## Abstract

**Objective:**

Temporal lobe epilepsy (TLE) is the most common cause of drug-resistant epilepsy and can be treated surgically to control seizures. In this study, we analyzed the relevant research literature in the field of temporal lobe epilepsy (TLE) treatment to understand the background, hotspots, and trends in TLE treatment research.

**Methods:**

We discussed the trend, frontier, and hotspot of scientific output in TLE treatment research in the world in the last 20 years by searching the core collection of the Web of Science database. Excel and CiteSpace software were used to analyze the basic data of the literature.

**Result:**

We identified a total of 2,051 publications on TLE treatment from 75 countries between 2003 and 2023. We found that the publication rate was generally increasing. The United States was the most publishing country; among the research institutions on TLE treatment, the University of California system published the most relevant literature and collaborated the most with other institutions. The co-citation of literature, keyword co-occurrence, and its clustering analysis showed that the early studies focused on open surgical treatment, mainly by lobectomy. In recent years, the attention given to stereotactic, microsurgery, and other surgical techniques has gradually increased, and the burst analysis indicated that new research hotspots may appear in the future in the areas of improved surgical procedures and mechanism research.

## Introduction

People of all ages, ethnicities, and socioeconomic levels are impacted by the chronic condition of epilepsy. It affects between five and ten people per 1000 people (1, 2). TLE is the more common type of epilepsy, with a variety of seizure forms that can manifest as sensory, emotional, motor, and psychiatric abnormalities. It originates in the temporal lobe and is characterized by focal aware seizure, focal impaired awareness seizure, or secondary generalized seizures. The two main types of TLE are medial temporal lobe epilepsy (MTLE) and lateral temporal lobe epilepsy (LTLE). MTLE is linked to changes in the hippocampal, parahippocampal gyrus, and amygdala. LTLE is a less common type in which seizures are restricted to the temporal lobe neocortex. With a frequency of 40% in epilepsy patients, TLE is the most prevalent kind of refractory focal epilepsy (3). Therefore, there has been much interest regarding the treatment of TLE.

In contrast to conventional literature reviews, bibliometric analysis using CiteSpace offers more insights (4). The knowledge map it creates is a part of the field of literature scientometrics, which can mine, analyze, and summarize the structure and process of knowledge growth in both temporal and spatial dimensions. It shows the development trend of a discipline or knowledge area within a certain period of time in an intuitive visual form and analyzes the evolution of multiple research frontiers. We sought to use CiteSpace to conduct a bibliometric analysis of the scientific literature on TLE treatment, to propose a scientometric methodology, and to provide and visualize research hotspots and trends related to TLE treatment. CiteSpace could help identify research areas that need further attention and enhance the clinical practice of TLE treatment. In addition, to fulfill the reader's expectations and avoid confusion, we state that, although this study is an econometric analysis of the scientific literature, no references will be provided.

## Data collection and research methods

### Data source and collection

On 2 April 2023, we searched the Web of Science Core Collection (WoSCC) for relevant English language articles in the field of TLE treatment with a time frame of 01-01-2003 to 31-03-2023. SCI-EXPANDED, CPCI-S, CPCI-SSH, BKCI-S, and BKCI-SSH were used as data sources, and the publication type was limited to “article.” The main search terms were “Temporal Lobe Epilepsies,” “Temporal Lobe Epilepsy,” “Lateral Temporal Epilepsies,” “Lateral Temporal Epilepsy,” and “Therapeutic, Therapy, Therapies, Treatment, Treatments.” In the Supplementary material, specific search tactics were given. The WoSCC database was searched independently by authors ZC and MG for pertinent material. The literature was then downloaded and saved in the format of “full record with cited references,” It was then inspected, and 2,051 documents were obtained as a sample for visual analysis.

### Research methods

CiteSpace, a Java-based bibliometric analysis visualization software developed by Prof. Chaomei Chen, is an interactive analysis tool for analyzing basic metrics of the literature, including country, journal, institution, keyword, and reference co-citation, capturing keywords with strong citation prominence, and constructing visual graphs (5). The version of CiteSpace software used in this study was 6.1.R6 (64-bit). CiteSpace was set up with the following parameters: performing time slices (1 year per slice) from January 2000 to March 2023, selecting all options in term sources, selecting one node type at a time, and selecting TOP50 as the standard, with other setting items as default values. Microsoft Office Excel 2016 (Microsoft, Redmond, Washington, USA) was used to process the data and generate statistical charts.

## Results

### Quantitative analysis of basic information

#### Statistics of publications

**Figure 1:**
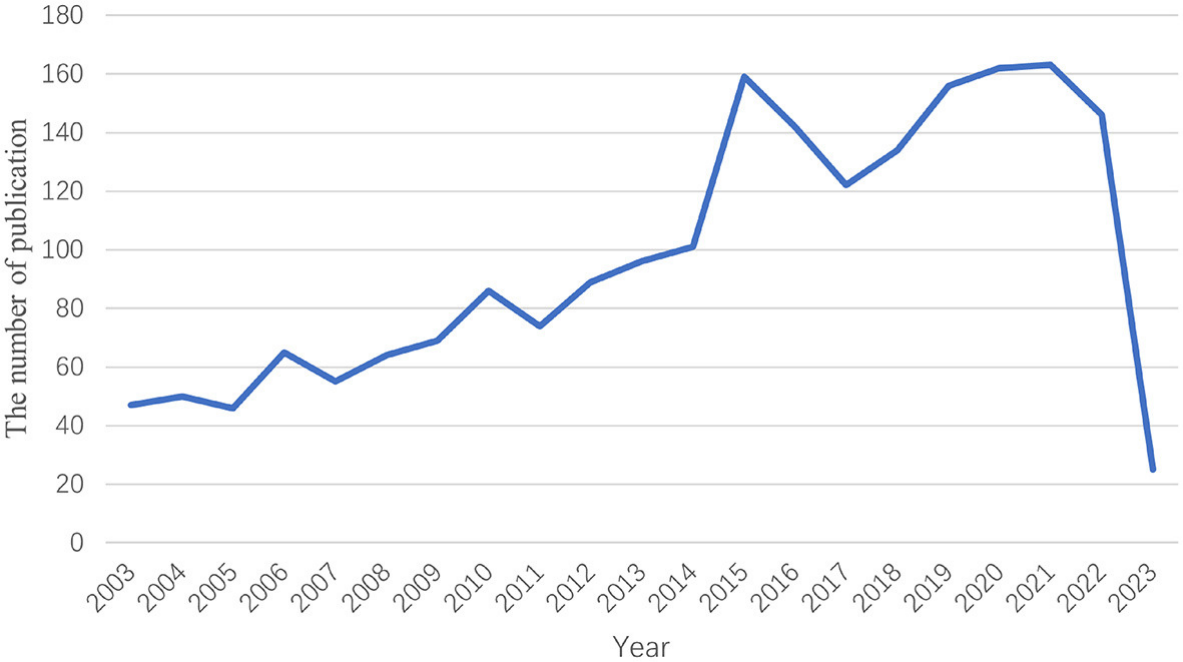
Time sequence of relevant studies on TLE treatment published from 2003 to 2023 in Web of Science.

In 1985, the International league against epilepsy (ILAE) proposed the third International Classification of Epilepsy Symptom Groups (ICSG). For the first time, partial epilepsies and epileptic syndromes were associated with relevant anatomical sites, and the concepts of frontal, parietal, and occipital lobe epilepsy were introduced. The fourth classification was made in 1989, further describing TLE (6). Since then, there have been more studies on TLE treatment. By searching the WoSCC database, a total of 2,051 articles on TLE treatment published between 2003 and March 2023 were obtained. As can be seen from Figure 1, although the annual number of publications on TLE treatment fluctuates, it still shows an increasing trend overall. These findings indicate that research on TLE treatment has become the focus of attention.

#### Analysis of countries/regions and institutions

**Figure 2:**
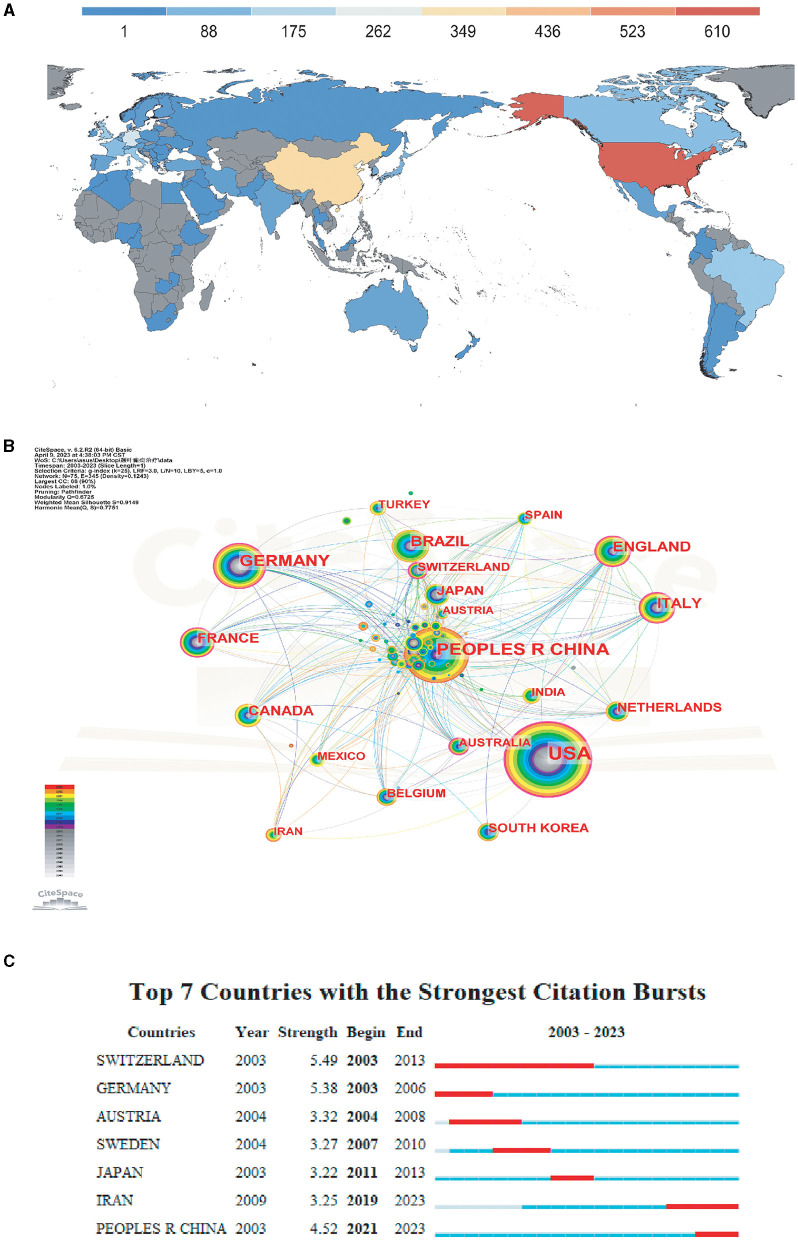
Analysis of countries engaged in research on TLE treatment. **(A)** Analysis of countries engaged in research on TLE treatment. **(B)** The distribution of countries in terms of publications. **(C)** The top 7 most productive countries.

We mapped the distribution of countries and regions (Figure 2A), and the resulting network had 345 linear connections between its 75 nodes. The node sizes correspond to the number of articles published in each country, while the connections between them showed the nature of the collaborative partnerships (Figure 2B). In the past 20 years, there were 75 countries/regions publishing publications on TLE treatment research, and the top 10 countries (5 European countries, 2 Asian countries, 2 North American countries, and 1 South American country) published a total of 1,181 articles, accounting for 57.58% of the total number of publications. The top three countries in terms of the number of publications were The United States (*n* = 610, 29.74%), followed by China (*n* = 351, 17.11%) and Germany (*n* = 220, 10.73%). The burst analysis (Figure 2C) indicated that TLE treatment research became a hotspot in Switzerland between 2003 and 2013, followed by an increase in China since 2021, and its popularity has continued until to date. In addition, there were extensive collaborations between many countries/regions, especially the US, not just with the country's neighbors, Canada and Mexico, but additionally with the UK, Italy, Belgium, Greece in Europe, Japan, and Taiwan (China) in Asia and Australia. Compared with The United States, China and other countries collaborated less, but there was also cooperation with Egypt, Mexico, the USA, Austria, Jordan, and other countries.

**Figure 3:**
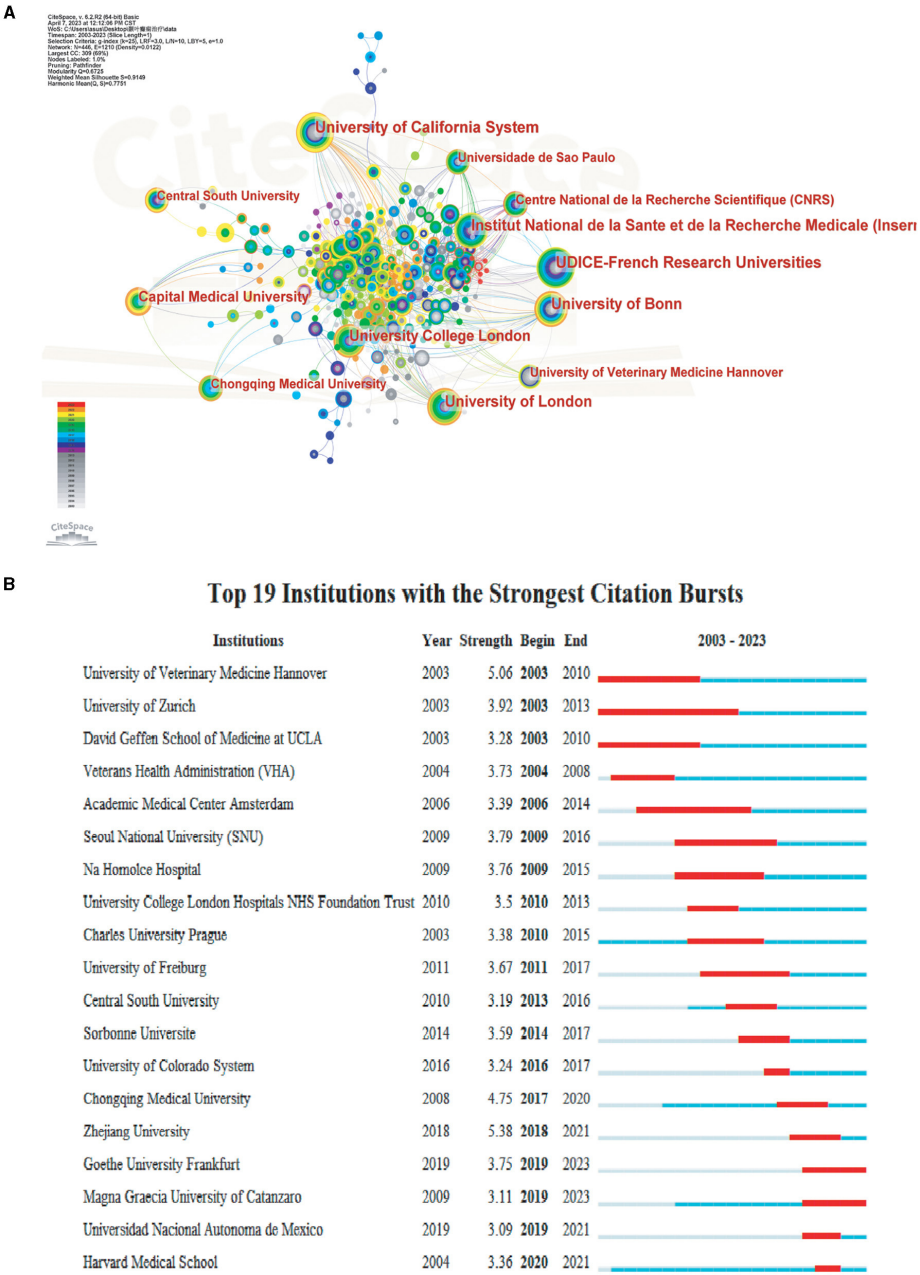
Analysis of institutions engaged in research on TLE treatment. **(A)** The distribution of institutions in terms of publications. **(B)** The top 19 most productive institutions.

Analysis of the collaborative network of research institutions in the literature related to TLE treatment (Figure 3A) showed that the issuing institutions were mainly universities. The top three institutions in terms of the number of publications were the University of California system (*n* = 80), UDICE-French Research Universities (*n* = 79), and the University of London (*n* = 67). In terms of the average time of publication, the institutions that published more studies generally had an earlier average year of publication, concentrated in the time period of 2003–2008, while the publications on TLE treatment from Chinese institutions were generally after 2008. Collaborations between institutions were extensive, with the University of California system having the most collaborations with other institutions, followed by University College London and University of London. The burst analysis revealed that famous foreign universities such as the University of Veterinary Medicine Hannover, University of Zurich, and Seoul National University (SNU) had higher burst intensity during 2003–2016, indicating that foreign institutions led the past of TLE treatment research. In recent years, the highest intensities were found in Zhejiang University, Zhejiang University, and Chongqing Medical University, showing the rapid development of TLE treatment research in China during recent years (Figure 3B).

#### Analysis of co-cited authors and co-cited journals

Two or more authors are cited in one or more articles at the same time. Then, these two or more authors are called co-cited authors. A total of 471 authors were co-cited, with the most co-cited author being ENGEL J (*n* = 442), followed by RACINE RJ (*n* = 382), WIEBE S (*n* = 322), KWAN P (*n* = 272), and LOSCHER W (*n* = 259), of which LOSCHER W, KWAN P, and WIEBE S were also high centrality authors (Table 1), indicating their outstanding contribution in this field.

**Table 1:** The top 5 Co-cited authors involved in research on TLE treatment.

**Rank**	**Count**	**Centrality**	**Year**	**Co-cited authors**
1	442	0.03	2003	Engel J
2	382	0.07	2003	Racine RJ
3	322	0.12	2003	Wiebe S
4	272	0.14	2003	Kwan P
5	259	0.15	2003	Loscher W

The top journal by number of citations was EPILEPSIA (*n* = 1,841), followed by EPILEPSY RESEARCH (*n* = 1,264), NEUROLOGY (*n* = 1,195), BRAIN (*n* = 990), and JOURNAL OF NEUROSCIENCE (973) (Table 2). The most cited article was published by Engel J et al. in JAMA-JOURNAL OF THE AMERICAN MEDICAL ASSOCIATION in 2012. This article concluded that surgery was superior to medication for MTLE, emphasizing the importance of early surgery (7).

**Table 2:** The top 5 Co-cited Journals involved in research on TLE treatment.

**Rank**	**Count**	**Centrality**	**Year**	**Co-cited Journal**
1	1841	0.02	2003	Epilepsia
2	1264	0.02	2003	Epilepsy Res.
3	1195	0	2003	Neurology
4	990	0.01	2003	Brain
5	973	0.03	2003	J. Neurosci.

### Exploring the hotspots and evolution of TLE treatment research based on literature co-citation

#### Co-citation literature co-occurrence analysis

**Figure 4:**
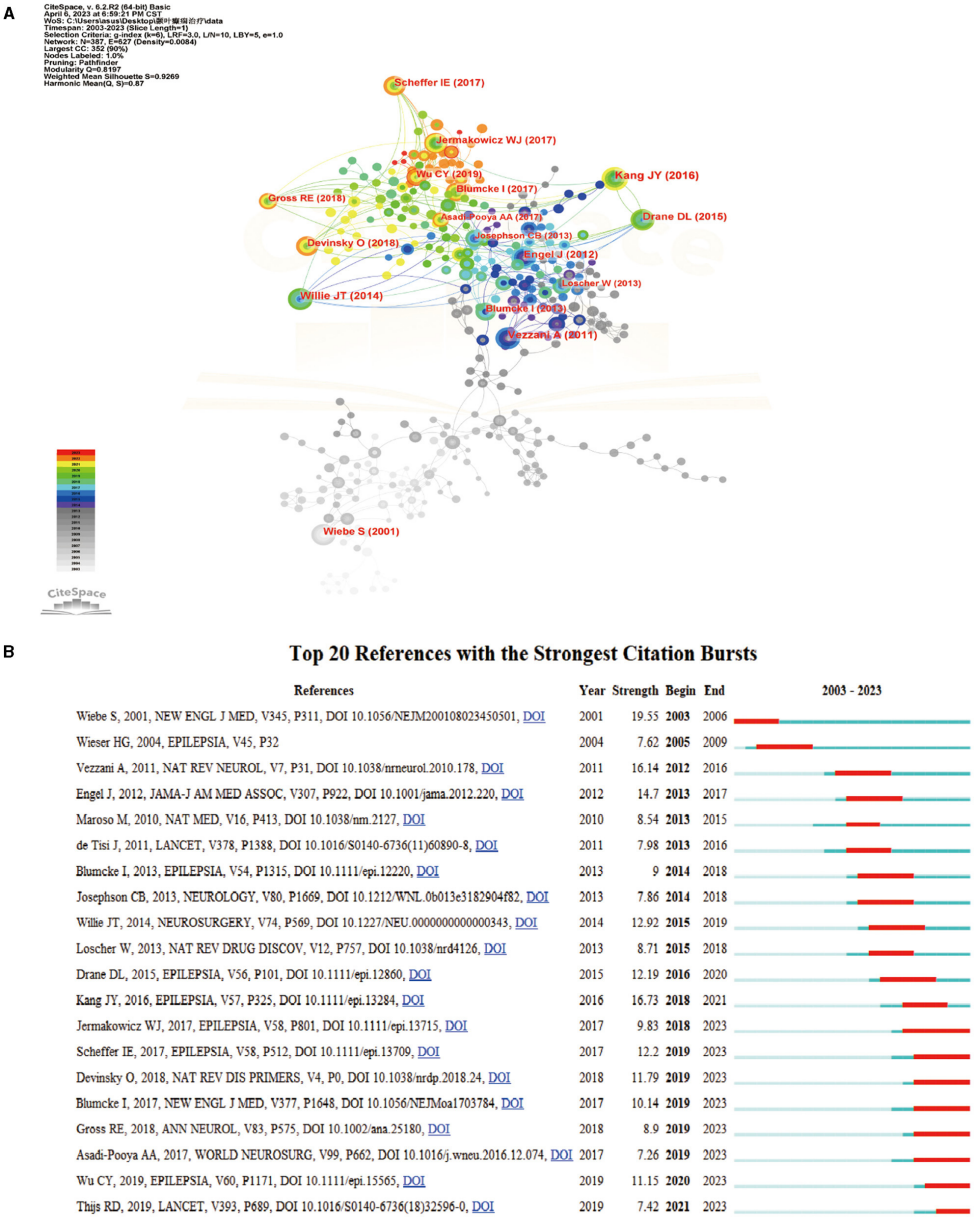
Analysis of co-cited references. **(A)** Network diagram showing cited references links. **(B)** References with periods of burst from 2003 onward among the top 20 burst references in articles related to TLE treatment.

Two articles are said to be in a co-citation relationship when they are cited by one or more studies concurrently, which is referred to as literature co-citation. The graph's nodes can be linked to represent this relationship, which can be mined for common concerns in the literature and a measure of closeness between two articles (8) (Figure 4A). For statistical purposes, we selected the top 10 co-cited articles in the time slice of 1 year and constructed the co-citation network graph. The number of citations was counted to obtain the ranking of citations (Table 3). Among them, the most co-cited article was published in EPILEPSIA by Kang et al. (9), followed by Vezzani et al. in Nat. Rev. Neurology, in addition to Willie et al. (11) in Neurosurgery in 2014 and Engel et al. (7) in JAMA-Journal of the American Medical Association in 2012. In the following, we will briefly analyze the articles with high co-citation frequency.

**Table 3:** Ranking of the number of cited references.

**Rank**	**Count**	**Centrality**	**Year**	**Cited references**
1	42	0.15	2016	Kang et al. (9)
2	38	0.13	2011	Vezzani et al. (10)
3	37	0.33	2014	Willie et al. (11)
4	35	0.09	2012	Engel et al. (7)
5	34	0.07	2015	Drane et al. (12)
6	33	0.3	2001	Wiebe et al. (13)
7	31	0.14	2017	Jermakowicz et al. (14)
8	30	0.05	2017	Scheffer et al. (15)
9	29	0.03	2018	Devinsky et al. (16)
10	25	0.21	2017	Blumcke et al. (17)

Surgical treatments for TLE continue to improve as technology evolves. In Engel et al. (7) conducted a comparative clinical study of continuous antiepileptic therapy vs. standardized anterior temporal lobectomy (ATL) plus antiepileptic therapy. In the second year of follow-up, none of the 23 participants in the medical group and 11 of the 15 participants in the surgical group were seizure-free. Thus, in patients with newly refractory disabling MTLE, excision plus antiseizure medication (ASM) was less likely to result in seizures in the second year of follow-up than ASM alone (7). Patients were usually referred for surgery 20 years after the seizure, but it was often too late to prevent severe disability and premature death. Engel et al. (7) reinforced the view that a surgery soon after the failure of two ASM trials offered the best chance of preventing a lifetime of disability (18–20). Subsequently, in Willie et al. (11) also concluded in a comparative clinical study that real-time MR-guided SLAH is a technically novel, safe, and effective alternative to open surgery (11). Due to the risk of neurocognitive deficits associated with open surgery for TLE, in Kang et al. (9) prospectively tracked seizure outcomes in 20 patients with drug-resistant surgical outcomes were evaluated at 2 months, 1 year, 2 years, and the most recent visit after surgery, and LiTT was found to have significant advantages over ATL. This led to the conclusion that MRI-guided stereotactic LiTT is a safe replacement for ATL (9). Previously, there had been limited reports on the use of LiTT in adult mTLE patients, with only two published case series studies with limited follow-up (11, 21). In contrast, this literature reported seizure outcomes in 20 patients with refractory mTLE who underwent MRI-guided LiTT, including an analysis of the effect of ablation volume on seizure outcomes and verbal memory performance.

In addition to the surgical treatment studies of TLE mentioned above, Vezzani et al. (10, 22, 23) investigated the connection between inflammation and epilepsy, how seizures trigger inflammation, if this subsequently occurring inflammation affects the frequency and severity of seizures, and the death of neurons associated with seizures. This investigation resulted in new molecular targets for the development of antiepileptic drugs.

#### Burst analysis of co-cited literature

The burst detection can present burgeoning trends in research and identify articles with a strong impact on future research in a timely manner (24, 25). The collected literature was screened, and the top 20 bursting literature was displayed according to the burst intensity (Figure 4B).

In terms of burst intensity, the highest burst intensity was found in the article published in New England Journal of Medicine by Wiebe et al. (13). They conducted a randomized controlled trial on 80 patients with TLE, and after 1 year, the patients in the surgical group had less awareness of seizure disorders and significantly better quality of life than the medical group. Therefore, it was concluded that surgical treatment was superior to long-term pharmacological treatment (13). One previous failed attempt at a randomized trial of surgery for epilepsy and commentators' emphasis on the difficulties inherent in executing such trials strengthened the view that they are not feasible (26, 27). However, the successful performance of this study demonstrated that randomized, controlled trials of surgery for epilepsy were feasible when the methods were adapted to the specific social and healthcare context of the patients. This study provided a reliable and precise estimate of the effectiveness and safety of surgery in patients with TLE. The second-highest burst intensity article was published in Epilepsia by Kang et al. (9). Kang et al. (9) also concluded that MRI-guided stereotactic LiTT was a safe alternative to ATL through a clinical trial study. The top two articles, in terms of burst intensity, both focused on the surgical treatment of TLE, indicating that surgical treatment occupies a major position in TLE treatment research (28). The articles published by Jermakowicz et al. (14) and Wu et al. (29) explored how to improve LiTT based on the article by Kang et al. (9). The article of Jermakowicz et al. (14) further explored the ablation and trajectory characteristics associated with optimal seizure control and attempted to minimize the risk of neurocognitive deficits in patients receiving TLE (mTLE) in LiTT treatment. However, the great variability of human anatomy made it difficult to determine the optimal LiTT trajectory and ablation (30). In the article, in addition to manual tracing for volumetric analyses, the authors were the first to apply non-rigid, deformable brain atlases to the study of neural ablation procedures. Wu et al. (29) used a new image-based method to assess the impact of surgical targets on the outcome of mTLE seizures in LiTT. They concluded that ablation needed to concentrate on the amygdala and also focus on the hippocampus head, parahippocampal gyrus, and nasal cortex to optimize the likelihood of no seizures. This study had several advantages: (1) a novel and robust methodology using nonlinear normalization and statistical modeling was presented to study complex brain therapies such as LiTT for mTLE; (2) it was applied to pre-existing data in the largest and most comprehensive multicenter study to date; and (3) the first three-dimensional model of a favorable laser ablation zone was presented (29). There were also articles by Willie et al. (11), Gross et al. (31), and Drane et al. (12) that demonstrated that MRI-guided stereotactic laser amygdala hippocampal dissection (SAH) was safer and more effective than open surgery. Engel et al. (7) in JAMA-Journal of the American Medical association also demonstrated that, in patients with new-onset intractable disabling mTLE, resection plus ASM treatment was less likely to result in seizures in the second year of follow-up compared with ASM treatment alone (7). de Tisi et al. (32) studied the long-term results of various TLE surgery techniques, suggesting room for further improvement in preoperative evaluation and surgical treatment of patients with chronic epilepsy (32). Josephson et al. (33) concluded that standard ATL improved the chance of freedom from disabling seizures in TLE patients by comparing standard ATL and SAH for seizure control after TLE (33). Moreover, the abovementioned articles were also at the top of the list for burst intensity.

Besides the above list of articles, of high burst intensity was the article by Vezzani et al. (10) in Nat. Rev. Neurology, which further discovered novel molecular targets for the development of antiepileptic medicines by examining the complicated function of inflammation in the development and progression of epilepsy. They focused on the rapidly growing body of evidence that supported the involvement of inflammatory mediators (released by brain cells and peripheral immune cells) in the origins of epileptic seizures and epileptogenic processes in individuals. Further understanding of the complex role of inflammation in the generation and progression of epilepsy generated new molecular targets for the design of antiepileptic drugs that could not only inhibit the symptoms of this disease but also prevent or abrogate the pathogenesis of the disease (10). Maroso et al. (34) explored the possibility that immune-related HMGB1-TLR4 signaling transduction might help with the generation and persistence of epileptic seizures and might target currently drug-resistant epilepsy to obtain anticonvulsant effects. Loscher et al. (35) discussed how outdated preclinical models and clinical investigation patterns have impeded the discovery of better treatments. They concluded that the development of future antiepileptic drugs might be improved through a new collaborative effort between higher education and business, through the recognition and implementation of tools for new aim-driven methods, through compared preclinical demonstration of concept studies, and through innovative clinical trial designs (35). It is indicative that investigators are continuing to explore pharmacotherapeutic targets for TLE beyond the surgical treatment of TLE.

In terms of burst time, in the past 20 years, research on TLE treatment has mainly revolved around surgical treatment, which in turn has undergone a shift from open surgery such as ATL and SAH to minimally invasive procedures such as LiTT. This indicates that, with the development of stereotactic and microsurgical techniques, the procedures for TLE treatment are also evolving with the times.

### Keywords-based analysis of hotspots and evolution of TLE treatment research

#### Keyword co-occurrence analysis

**Figure 5:**
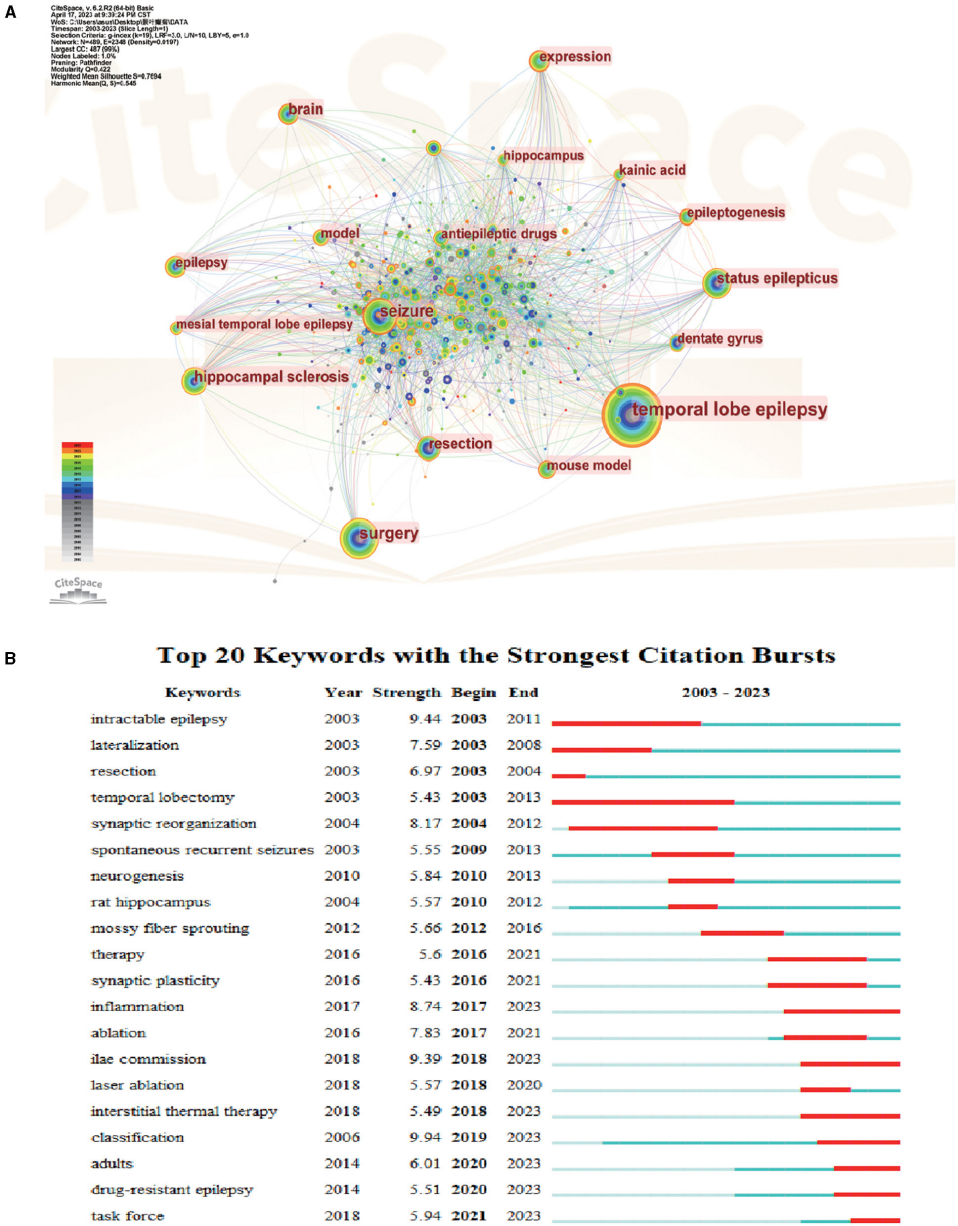
Analysis of keyword cluster map on TLE treatment. **(A)** A CiteSpace visualization map of keywords analysis. **(B)** Keywords with periods of burst from 2000 onward are among the top 20 burst keywords in articles related to TLE treatment.

Keywords are refined expressions of literature topics, and their frequency of occurrence is often strongly associated with the research hotspots in the field. In this study, keyword co-occurrence analysis was performed on 2,051 studies from January 2003 to March 2023, and the time slices were set to 1 year after combining similar keywords (seizures to seizure, rats to rats, rat model to mouse model, epilepsy surgery to surgery, surgical treatment to surgery, lobectomy to resection). The keyword co-occurrence map (Figure 5A) was created by choosing the top 30 keywords that appeared the most frequently in each area. The size of each node, which stands for a keyword, reflects how frequently the keyword occurs together. The nodes' and connecting lines' color corresponds to the passage of time: from far away to close at hand, the color changes from dark to light. The ten most frequently used keywords in the top 10 were temporal lobe epilepsy, surgery, seizures, hippocampal sclerosis, status epilepticus, resection, brain expression, kainic acid, and dentate gyrus. Among them, except for temporal lobe epilepsy, surgery had the highest frequency, and resection was also at the top of keyword co-occurrence, which indicated that surgery had an important position in the field of TLE treatment. The goal of epilepsy surgical treatment was to apply neurosurgical techniques to terminate seizures or to decrease the frequency and/or intensity of seizures while maximizing neurological function and focusing on improving the patient's quality of life. Some studies showed that temporal lobe surgery had a 60–80% chance of completely controlling seizures (4). Hippocampal sclerosis, antiepileptic drugs, children, and animal models were keywords with a large centrality, indicating that these words were the key nodes in the graph. This indicated that researchers were actively exploring the surgical treatment of TLE and also studying the efficacy and prognosis of antiepileptic drug treatment through clinical studies and the establishment of animal models. In addition, the treatment of TLE in children has received much attention.

#### Keyword cluster analysis

The clustering effect of CiteSpace software mapping can be measured by the Q and S values, and according to general consensus, Q > 0.3 indicates a strong clustering structure, S > 0.5 indicates good clustering, and S > 0.7 indicates a compelling and effective clustering. Keyword clustering analysis could help to discover the distribution of relevant research in a field, and this study used the log-likelihood ratio (LLR) test algorithm to cluster the keywords. The smaller the cluster number, the larger the research size and the higher the research intensity of the literature under that cluster. The keyword clustering was calculated by keyword co-occurrence mapping to obtain Table 4. As shown in Table 4, the first 8 clusters were all ≥ 8 in size and had high profile coefficients, indicating successful clustering and high confidence.

**Table 4:** Main clusters of keywords.

**Cluster ID**	**Size**	**Silhouette**	**Mean year**	**Label (LLR, log-likelihood ratio)**
1	117	0.805	2008	Epilepsy surgery (202.66, 1.0E-4); anterior temporal lobectomy (61.15, 1.0E-4); mesial temporal sclerosis (57.99, 1.0E-4); mesial temporal lobe epilepsy (54.91, 1.0E-4); and temporal lobectomy (53.91, 1.0E-4)
2	97	0.71	2010	Mossy fiber sprouting (56.16, 1.0E-4); epilepsy surgery (50.09, 1.0E-4); dentate gyrus (46.74, 1.0E-4); kainic acid (38.46, 1.0E-4); neurogenesis (29.73, 1.0E-4)
3	65	0.765	2009	Status epilepticus (38.85, 1.0E-4); epilepsy surgery (29.74, 1.0E-4); and oxidative stress (23.85, 1.0E-4); epileptogenesis (20.37, 1.0E-4); and reactive oxygen species (15.82, 1.0E-4)
4	56	0.755	2010	Deep brain stimulation (50.68, 1.0E-4); DBS (21.1, 1.0E-4); epilepsy surgery (20.11, 1.0E-4); electrical stimulation (19.38, 1.0E-4); and antiepileptic drugs (19.2, 1.0E-4)
5	54	0.761	2012	Neuroinflammation (23.18, 1.0E-4); epilepsy surgery (22.27, 1.0E-4); expression (18.69, 1.0E-4); TRH (13.19, 0.001); and dreadd (13.19, 0.001)
6	47	0.704	2013	Drug-resistant epilepsy (17.59, 1.0E-4); personality (15.35, 1.0E-4); status epilepticus (14.43, 0.001); focal epilepsy (13.88, 0.001); and depression (11.31, 0.001)
7	42	0.781	2013	Functional connectivity (41.56, 1.0E-4); graph theory (25.56, 1.0E-4); voxel-based morphometry (20.74, 1.0E-4); fmri (17.88, 1.0E-4); and atrophy (15.82, 1.0E-4)
8	9	0.962	2016	Hallucinations (22.43, 1.0E-4); delusions (22.43, 1.0E-4); antipsychotics (22.43, 1.0E-4); psychosis (16.9, 1.0E-4); and neurocysticercosis (11.16, 0.001)

The main label contents explored by the clustering module were extracted, and clusters #0, #2, #3, and #6 were mainly studies of TLE surgical treatment; clusters #1 and #4 were experimental studies of TLE mechanisms and drugs; clusters #5 and #7 were studies of TLE-related neurological and psychiatric diseases; chronologically, cluster #0 was the earliest, indicating that researchers began to focus on TLE surgical treatment and explore the effects and prognosis of surgical treatment at an earlier time; cluster #7 was the latest, indicating that studies of TLE-related psychiatric treatment have been a hotspot in the field of TLE treatment in recent years.

#### Keyword burst analysis

Keyword burst analysis can clarify the distribution and evolution of the research focus and hot research topics in a certain time period. The results of keyword burst analysis are shown in Figure 5B. According to the burst table, the literature could be roughly divided into three research phases: from 2003 to 2010, the five hot keywords that appeared frequently were “intractable epilepsy,” “lateralization,” “resection,” “temporal lobectomy,” and “synaptic reorganization,” indicating that lobectomy was the main treatment for TLE in this period; from 2010 to 2016, the keywords of this phase were “spontaneous recurrent seizures,” “neurogenesis,” “rat hippocampus,” “mossy fiber sprouting,” the therapeutic research of TLE in this phase was to investigate the mechanism of TLE occurrence by establishing animal models of epilepsy, mostly concerning mossy fiber sprouting and neurogenesis, aong others; from 2016 to 2023, the keywords in this phase were richer and mainly focused on “therapy,” “synaptic plasticity,” “ablation,” “laser ablation,” “inflammation,” “ilae commission,” “interstitial thermal therapy,” “classification,” “results,” “drug-resistant epilepsy,” and “task force.” The keywords in this phase indicated that the research of TLE treatment in this phase was divided into two aspects: on the one hand, studying the mechanism of TLE, such as synaptic remodeling and inflammatory factors involved; on the other hand, conducting the clinical trials to find improved approaches to TLE surgical treatment, such as laser thermotherapy. New treatments, such as LiTT, have appeared, while ATL continues to be the “gold standard” for treating drug-resistant mTLE. Owing to its minimally invasive nature, LiTT is associated with a brief hospital stay, reduced mortality, and lower instances of cognitive impairment (29, 36).

## Discussion

To our knowledge, this study was the first bibliometric evaluation to concentrate on TLE therapy research. The significant majority of publications in the field of TLE therapy were covered by the data we collected from the WoSCC database, and the data analysis was quite objective and thorough, clearly illustrating the state of TLE treatment research at the time. Inevitably, however, there were some limitations to this study. First, this study consisted of original articles published between 2003 and 2023 and indexed in the WoSCC database, which might have excluded relevant studies that were not included in the review. Therefore, the literature included in our study might not be exhaustive. Second, because the analysis focused on English-language studies, relevant studies in other languages might have been overlooked. Moreover, due to the low citation rate, recently published high-quality articles might not have received the attention they deserve, indicating the importance of updating future studies. It was even more important to emphasize that our analysis relied on bibliometric data and lacked information on the clinical effectiveness of the treatments reviewed. Since they included the vast majority of studies in the field of TLE treatment research, the articles included in the study—while they may not accurately reflect all research on TLE treatment—were adequate for analyzing tendencies and hotspots in the field.

### Basic information about the research

In this study, articles on TLE treatment published between 2003 and 2023 were found through an organized search of the Web of Science database. This scientometric analysis included 2,051 English-language publications from 75 different nations after discarding studies that did not adhere to the screen standards. We used CiteSpace software to quantitatively and visually assess findings from studies and developments in the field, exploring research hotspots and trends in TLE treatment. Regarding the number of publications, even if the annual publishing count has changed a little over the previous 20 years, the overall trend in the number of yearly publications was found to be increasing based on polynomial fitting curves. This finding indicated that an increasing number of scholars were interested in TLE treatment. The findings suggested that the articles in this category were both experimental and clinical studies, mainly describing the pathogenesis, effectiveness of surgical treatment, and prognosis of TLE. The articles establish a foundation for the creation of medications and surgical techniques for TLE treatment. In terms of nations, the United States, China, Germany, and Brazil were among the first to study TLE treatment, and the United States had the most studies in this area. In the analysis of institutional bursts, overseas institutions had previously led the growth in this field, but Chinese institutions showed the greatest burst intensity in recent years, showing the rapid advancement of China.

### Research hotspots in the TLE treatment field

#### Surgical treatment

Surgical resection is the gold standard of care for drug-resistant focal epilepsy (11). The tendency in epilepsy surgery is to minimize the volume of tissue removed and search for fewer invasive surgical methods to ablate epileptic lesions (19).

A relatively new technique was LiTT, which was a minimally invasive stereotactic approach. Advantages of LiTT over other minimally invasive procedures, such as stereotactic radiosurgery, included real-time visualization of ablation via MRI thermometry, discrete lesion margins, and immediate results. Instantaneous feedback allowed immediate confirmation of the stereotactic trajectory and tight control over the size of the ablation volume. From the patient's perspective, LiTT had the added benefit of a much lower risk of infection and a much shorter recovery time (9, 11).

In addition, treatments such as brain-responsive stimulation and computerized surgical resection might be new treatment options (2, 37). It can be observed that, with the development of stereotactic and microsurgical techniques, the surgical approach to TLE treatment is evolving (38).

#### Research on the TLE mechanism

Research on TLE mechanisms revolves around mossy fiber outgrowth, dentate gyrus, neurogenesis, and neuroinflammation.

Hippocampal sclerosis and axonal rearrangement are two histological abnormalities that are frequently observed in TLE. The dentate gyrus (DG) experiences mossy fiber sprouting (MFS), which is the most frequent rearrangement following epileptogenesis. Dentate granule cell axons that branch out to CA3 hippocampal pyramidal cells are known as mossy fibers. Sustained epilepsy (SE) leads to the formation of new synaptic connections (sprouting) between mossy fibers and granule cell dendrites (39, 40). Studies have shown that the severity of MFS correlates with the frequency of spontaneous recurrent seizures (41).

Seizure activity induces neurogenesis in the hippocampus. Neonatal cells express proteins associated with brain plasticity and are involved in cell migration, synaptogenesis, axon growth, and branching (42). Whereas, neuroinflammation is directly involved in epileptogenesis and neurodegeneration, promoting chronic epilepsy and cognitive deficits. SE causes hippocampal damage by inducing inflammation, and hippocampal damage eventually leads to chronic TLE. Therefore, epilepsy-induced neuronal damage can be ameliorated through anti-inflammatory effects (22, 43–45). Through the continuous study of the TLE mechanism, we can find the target of TLE treatment.

#### TLE-related neurological and psychiatric disorders

The existence of psychological conditions among epileptic patients may reflect a multifactorial interaction between pre-seizure dysfunction and/or epilepsy-related psychosocial, medical, neurophysiological, and neurochemical changes and the neurological damage that leads to epilepsy. The high prevalence of psychiatric disorders has long been recognized in MTLE patients (46). Indeed, several causative mechanisms of mood and anxiety disorders have been found to contribute to the development of epilepsy (47), such as endocrine disorders, inflammatory mechanisms, and neurotransmitter disorders (48–50).

After TLE surgery, it has also been observed that psychiatric symptoms can appear for the first time or for pre-existing symptoms to worsen. The most common postoperative psychiatric disorders include depression, anxiety, and, less commonly, psychosis (51). Vermoesen et al. (52) also found that antidepressant doses of both citalopram and reboxetine reduced the frequency of spontaneous seizures in epileptic rats (52).

Further exploration of TLE-related psychiatric disorders will help the surgical decision-making process and enhance postoperative care. In addition, these studies may also contribute to our understanding of the pathogenesis of postoperative psychopathology (51).

### Research hotspot prediction

#### Surgical treatment mainly by laser ablation

Although ATL is still the “gold standard” for the treatment of drug-resistant mTLE, currently, there are novel treatments, such as LiTT. Being a minimally invasive procedure, LiTT has a shorter hospital stay, a lower incidence, and less damage to cognitive function (9, 29). The heat of LiTT continues to this day, indicating that laser ablation-based surgery is a research hotspot in the future.

#### Study on mechanisms related to synaptic remodeling and inflammation

Researchers have explored the related mechanisms of TLE. The heat continues to this day, mainly related to synaptic remodeling and inflammation, which provides a new strategy and direction for the modernization of TLE drug treatment in the future.

The most typical comorbidity of TLE is cognitive impairment. Approximately 50% of epilepsy patients are cognitively impaired in one or more ways. Synaptic plasticity is a molecular mechanism of learning and memory. Impairment of synaptic plasticity plays a crucial role in the cognitive comorbidity of TLE (53, 54). Inflammatory brain processes are extensively implicated in epileptogenesis (4). For instance, numerous studies have demonstrated that TLE patients' brains contain an active immune system (55).

CiteSpace first calculates the visualization network of TLE treatment references. Based on the results of CiteSpace, the research hotspots and trends in this field were established. Through the cluster analysis of co-cited literature and keywords, we obtained the research hotspots of TLE treatment as surgical treatment, mechanism research, and related mental and neurological diseases. In addition, we investigated the emergence analysis of co-cited literature and keywords and predicted that the research trends of TLE treatment are surgical treatment based on laser ablation, mechanism research related to synaptic remodeling, inflammation, and so on. This study demonstrated a bibliometric analysis methodology, the results of which visualize the research hotspots and trends to understand the convenience of peer researchers.

## Conclusion

Based on the bibliometric method, this study used CiteSpace and WOS databases to visually analyze the literature on TLE treatment research in terms of references, institutions, keywords, etc. We could identify the primary research questions and broad trends in this area with the aid of information visualization, and we could share the data we've gathered with upcoming researchers. The findings revealed that, throughout the previous 20 years, the research in this field has been greatly developed, and the focus of TLE treatment research has also changed. The improvement of surgical procedures, mechanism research, and TLE-related neurological and mental diseases are the current mainstream research hotspots. Improving surgical procedures to reduce the complications and prognosis of surgical treatment and the study of TLE mechanism may become the future research trend, which provides a theoretical basis for the future research direction of TLE treatment and promotes the development of new means of TLE treatment.

## Data availability statement

The original contributions presented in the study are included in the article/Supplementary material, further inquiries can be directed to the corresponding author.

## Author contributions

ZC, MG, and XC contributed to all tables and figures and the main manuscript. ZC and MG downloaded and collated the relevant studies. HL conceived of the study and provided the methodological guidance. SH, YX, and MZ revised and supplemented the manuscript. All authors contributed to the article and approved the latest version.
