# Supplementary material for: Progress in TLE treatment from 2003 to 2023: scientific measurement and visual analysis based on CiteSpace
Source: Front Neurol. 2023 Oct 3;14:1223457. doi: 10.3389/fneur.2023.1223457 (PMC10580429; doi:10.3389/fneur.2023.1223457)
Supplement: Supplementary file 1 [file Table_1.DOCX]

Search strategy:

Databases: SCI-EXPANDED, CPCI-S, CPCI-SSH, BKCI-S, BKCI-SSH

#1: TI= ("Temporal Lobe Epilepsies") OR TI= ("Temporal Lobe Epilepsy") OR TI= ("Lateral Temporal Epilepsies") OR TI= ("Lateral Temporal Epilepsy") OR AB= ("Temporal Lobe Epilepsies") OR AB= ("Temporal Lobe Epilepsy") OR AB= ("Lateral Temporal Epilepsies") OR AB= ("Lateral Temporal Epilepsy")

#2: TI=(Therapeutic) OR TI=(Therapy) OR TI=(Therapies)OR TI=(Treatment)OR TI=(Treatments)OR AB=(Therapeutic) OR AB=(Therapy)OR AB=(Therapies) OR AB=(Treatment) OR AB=(Treatments)

((#1 AND #2) AND DT=(Article)) AND LA=(English)

DOP: 2003-01-01 to 2023-03-31
